# High‐dose atorvastatin improves vascular endothelial function in patients with leukoaraiosis

**DOI:** 10.1002/jcla.23081

**Published:** 2020-03-10

**Authors:** Jianan Xue, Zhisheng Wu, Shujie Gong, Shengying Qin, Aiming Gu

**Affiliations:** ^1^ Department of Clinical Laboratory Jingjiang Chinese Medicine Hospital Jingjiang China; ^2^ Department of Neurology The First Hospital of Quanzhou Affiliated to Fujian Medical University Quanzhou China; ^3^ Bio‐X Institutes Shanghai Jiao Tong University Shanghai China; ^4^ Department of Neurology Jiaxing Maternity and Child Health Care Hospital Jiaxing China

**Keywords:** atorvastatin, leukoaraiosis, reactive hyperemia index, vascular endothelial function

## Abstract

**Objective:**

Leukoaraiosis (LA), as an age‐related white matter degeneration, is mainly caused by chronic ischemia. Our study aims to explore the efficacy of different doses of atorvastatin (ATV) in the vascular endothelial function in patients with LA.

**Methods:**

Our study enrolled 402 LA patients who were then randomly included as control or treated with ATV (10 mg), ATV (20 mg), or ATV (30 mg). The total cholesterol (TC), triglycerides (TG), high‐density lipoprotein cholesterol (HDL‐C), and low‐density lipoprotein cholesterol (LDL‐C) were detected by enzyme colorimetric assay. The high‐sensitivity C‐reactive protein (hs‐CRP) level, reactive hyperemia index (RHI), endothelin‐1 (ET‐1) content, and nitric oxide (NO) level were tested by latex agglutination test, peripheral arterial tonometry technology, radioimmunoassay, and nitrate reductase assay, respectively.

**Results:**

After 8 weeks of ATV treatment, the levels of TC, LDL‐C, and HS‐CRP decreased significantly, and the trends were demonstrated in a more significant way with the increases of dose of ATV. The treatment with ATV at different doses elevated NO level and RHI and declined ET‐1 content. Gastrointestinal reaction, muscular pain, and increased aminopherase were observed after treatment with the ATV at different doses with more obvious symptoms detected accompanied by the increase of the dose. The RHI was in negative correlation with the ET‐1 and HS‐CRP while in positive correlation with NO.

**Conclusion:**

Our study demonstrates that ATV can significantly improve the vascular endothelial function in LA patients with a dose‐dependent effect.

## 1 INTRODUCTION

Leukoaraiosis (LA), or severity of white matter hyperintensity, is a crucial biomarker for cerebrovascular disease.^1^ As an ischemic white matter lesion, LA is associated with poor post‐stroke outcomes and an increased stroke risk^2^ and refers to an age‐related presence of the brain white matter on neuroimaging.^3^ LA is closely related to ischemic stroke, dementia, and intracerebral hemorrhage.^4^ One study specified that structural vascular abnormalities featured by vessel wall thickening were linked to LA, verifying the assertion that LA was generated by vascular changes and ischemia.^5^ LA predominantly influences the subcortical white matter, evidently indicating a correlation with the cortical microvascular dysfunction, and potentially declined cortical ischemic tolerance.^6^ Endothelial dysfunction, characterized by declined nitric oxide (NO) bioavailability, was present as the first stage during the development of coronary artery disease.^7^ Vascular endothelial function, decreased with aging, is closely related to an elevated risk of cardiovascular disease, and particularly aerobic exercise, lifestyle modification as well as dietary adjustment had a favorable effect on vascular aging,^8^ while the assessment of the vascular endothelial function lacks consistency.^9^ Interestingly, atorvastatin (ATV) may enhance endothelial function for relatively moderate nicotine‐dependent smokers.^10^

Atorvastatin, as a synthetic inhibitor of 3‐hydroxy‐3‐methylglutaryl‐coenzyme A, presents with a long plasma half‐life and lipid‐lowering ability and is commonly performed to reduce cholesterol levels.^11^ As an HMG‐CoA reductase inhibitor, ATV is often used in the treatment of hypercholesterolemia.^12^ In addition, it is widely used for the treatment of dyslipidemias and exhibits protective effects against seizures, and a study demonstrated its effect on oxidative stress markers and certain neurotransmitter and on animal models of anxiety, seizures, and depression.^13^ A high‐fat diet could lead to endothelial dysfunction related to inflammation, and ATV might be capable of counter‐regulating it.^14^ Another study explored the role of local and systemic ATV application on periodontium by using immunohistochemical and histomorphometric analyses and demonstrated its beneficial effects on periodontium after the induction of experimental periodontitis, indicating that ATV can also be treated as a therapeutic and protective marker for periodontal disease.^15^ A previous study investigated the effect of ATV therapy on disease activity, inflammation, arterial stiffness, and endothelial dysfunction in patients suffering from rheumatoid arthritis, and the results indicated that ATV therapy in patients with rheumatoid arthritis inhibited disease activity and vascular risk factors promoting the atheromatous lesion.^16^ Thus, this study aims to explore the role of ATV in the vascular endothelial function in patients with LA, so as to provide a therapeutic method for LA treatment.

## 2 MATERIALS AND METHODS

### 2.1 Ethical statement

The present study was performed with the approval of the Ethics Committee of Jiaxing Maternity and Child Health Care Hospital and all subjects signed written consents.

### 2.2 Study subjects

From a time period between July 2013 and January 2015, a total of 402 LA patients, confirmed by magnetic resonance imaging (MRI), were chosen for this study from Jiaxing Maternity and Child Health Care Hospital, among which 222 cases were male and 180 were female aged from 32 to 86 years old (mean age: 56.85 ± 9.74). Patients would be included the following: (a) all LA patients admitted in Jiaxing Maternity and Child Health Care Hospital were verified by MRI and were with complete clinical data, (b) all LA patients were in consistency with the inclusion standards referred by Wadia et al,^17^ (c) according to minimental state examination (MMSE) scoring: illiterate >17 points, primary school >20 points, and middle school or more >24 points; Mo‐CA score >26 points; activities of daily living scale (ADL) grade ≥22; frequently asked question (FAQ) score grade >9 points; clinical dementia rating (CDR) range from 0 to 0.5 points; and global deterioration scale (GDS) was in state 2 or 3. Patients would be excluded if (a) patients with diseases such as acute cerebral infarction, cerebral hemorrhage, subarachnoid hemorrhage, subdural hematoma, and others; (b) patients with cancer history, prolonged application of immunosuppressant drugs or chemotherapeutics; (c) patients with LA resulting from immunity, poisoning, multiple sclerosis, or hypothyroidism but blood vessel; (d) patients with acute or chronic inflammation, liver and renal failure, systemic lupus erythematosus, peripheral vascular disease, or thromboembolic disease; (e) patients who took lipid‐lowering drugs like statin in the latest half year; and (f) patients with statin‐like drug contraindication (active liver disease, allergy to statin drugs).

### 2.3 MRI and grading standards

Head MRI using 3.0 T superconducting MRI system (German Siemense Company) with eight‐channel phased‐array head coi was conducted for all patients. The T1‐weighted imaging (T1WI) was adapted (repetition time [TR] = 2633 ms, echo time [TE] = 23.4 ms, Matrix 128 × 97, 2 times), T2‐weighted imaging (T2WI, TR = 5000 ms, TE = 119 ms), and also T2 fluid‐attenuated inversion recovery (FLAIR) (TR = 9600 ms, TE = 114 ms, inversion time [TI] = 2400 ms, Matrix 320 × 192, field of view [FOV] = 240 mm × 240 mm, 5 mm in thickness with 1 mm as interval). The obtained images were evaluated and observed by two experienced doctors without acknowledging the clinical and experimental results before. A patient was confirmed with LA when symmetrical structure, unclear patchy fusion or non‐fusion area, relative low density of T1WI signal (relative low density of CT signal), and high density of T2WI signal and T2FLAIR were observed in periventricular white matter of two lateral ventricles. Foci exhibited low‐density signal in MRI and long T1 and T2 signals. All patients were divided into four levels according to the Aharon‐Peretz standard,^18^ and the concrete grading was presented as follows: LA‐1, low‐density area was observed on the frontal or occipital horn of lateral ventricle; LA‐2, low‐density area was observed on both the frontal and occipital horn of lateral ventricle; LA‐3, successive low‐density area was observed lateral ventricle with foci; LA‐4, foci were observed around the cella lateralis and corona radiate area.

### 2.4 Grouping and treatment regimens

Random number table was adapted for grouping, and patients were assigned into the control group and ATV groups which were further classified into ATV (10 mg) (67 cases, treated with 10 mg of ATV), ATV (20 mg) (67 cases, treated with 20 mg of ATV), and ATV (40 mg) (67 cases, treated with 40 mg of ATV) groups. The control group included 201 cases, among which 109 were male and 92 were female aged from 34 to 86 years old (mean age: 57.36 ± 10.13). Before selection, there were no significant differences in terms of age, gender, and disease history (Table 1), and there were no significant differences in parameters such as heart rate, blood pressure, and blood lipid (all *P* > .05). Patients in the ATV groups, aside from the regular treatment, were administered oral doses of different AVT concentrations (Lipitor, Pfizer Inc, 10, 20, and 40 mg, respectively) once every night for 8 weeks whereas the patients in the control group only received regular treatment. The diet and lifestyle of all subjects were maintained in the same routine during experiments. Before and after 8 weeks of treatment, venous blood samples were extracted on an empty stomach for detection of blood fat, levels of high‐sensitivity C‐reactive protein (HS‐CRP), endothelin‐1 (ET‐1), and nitric oxide (NO). In addition, endothelial peripheral arterial tonometry (Endo‐PAT) was used to detect and observe vascular endothelial function.

**Table 1:** Comparisons of clinical data of subjects among the control and ATV (10 mg), ATV (20 mg), and ATV (30 mg) groups

General conditions	Control group (n = 201)	ATV (30 mg) group (n = 67)	ATV (20 mg) group (n = 67)	ATV (30 mg) group (n = 67)
Male/female	109/92	35/32	40/27	38/29
Age	58.33 ± 8.70	56.22 ± 7.73	58.22 ± 8.99	57.03 ± 7.52
Heart rate	70.11 ± 4.36	69.63 ± 2.78	71.08 ± 3.55	70.36 ± 4.03
Systolic blood pressure	133.24 ± 10.83	134.59 ± 7.93	132.60 ± 8.33	135.21 ± 9.51
Diastolic blood pressure	80.14 ± 5.23	82.43 ± 4.27	79.91 ± 4.71	81.14 ± 5.21
Blood fat				
TC	5.45 ± 0.63	5.51 ± 0.59	5.57 ± 0.64	5.52 ± 0.71
LDL‐C	1.92 ± 0.17	1.90 ± 0.21	1.91 ± 0.22	1.93 ± 0.25
HDL‐C	1.01 ± 0.13	1.04 ± 0.15	0.99 ± 0.15	1.02 ± 0.11
TG	2.32 ± 0.26	2.36 ± 0.28	2.28 ± 0.31	2.37 ± 0.32
Disease history				
Hypertension	62 (31%)	20 (30.4%)	22 (33.1%)	25 (37.3%)
Diabetes	85 (42.1%)	25 (36.9%)	24 (35.4%)	25 (37.8%)
Smoke history	78 (38.6%)	25 (37.3%)	28 (41.3%)	31 (45.8%)
Coronary disease	38 (18.9%)	16 (23.5%)	14 (20.9%)	18 (26.3%)
LA grading				
LA‐1	98 (48.9%)	27 (39.9%)	29 (43.1%)	29 (43.3%)
LA‐2	65 (32.34%)	24 (35.82%)	19 (28.35%)	21 (31.34%)
LA‐3	23 (11.4%)	12 (18.4%)	13 (19.2%)	12 (17.9%)
LA‐4	15 (7.46%)	4 (5.97%)	6 (8.95%)	5 (7.46%)

Abbreviations: ATV, atorvastatin; HDL‐C, high‐density lipoprotein cholesterol; LA, leukoaraiosis; LDL‐C, low‐density lipoprotein cholesterol; TC, total cholesterol; TG, triglycerides.

### 2.5 Enzyme colorimetric assay

OLYMPUS AU400 automatic biochemical analyzer was employed. The total cholesterol (TC), triglycerides (TG), and high‐density lipoprotein cholesterol (HDL‐C) reagents and standards were obtained by Olympus Optical Co., Ltd. and ApoA and ApoB reagents and standards by Jiuqiang Bio Co., Ltd. All patients in the control and ATV groups were tested, with no drinking or high‐fat diet before detection. The venous blood samples extracted on an empty stomach were reserved in biochemical coagulation tubes; the serum was separated at 4000 r/min and detected within 2 hours. The TC, TG, HDL‐C, and LDL‐C were detected by enzyme colorimetric assay. All aforementioned indexes were detected within 24 hours of onset of LA and 8 weeks after ATV treatment on an empty stomach.

### 2.6 Detection of HS‐CRP

Heparin anticoagulant (3 mL) was extracted from a vein on an empty stomach from patients in the control, ATV (10 mg), ATV (20 mg), and ATV (40 mg) groups and was centrifuged for 5 minutes (4000 r/min) to separate blood plasma, after which it was then reserved in a refrigerator at 38°C for further use. HS‐CRP in blood plasma was detected by latex agglutination test (BN100; Baldor Electric Company).

### 2.7 Reactive hyperemia index (RHI) detection

The Endo‐PAT technology (AUDEN) was employed for RHI detection. The principle was based on the biosensor system, in combination with the peripheral arterial tension measurement (PAT) for detecting the change in fingertip pulse volume (blood flow). All patients while lying flat wore pneumatic cuff in the right arms and a probe containing sensor and pneumatic appliance was attached on the forefinger. After inflation, the blood flow signals were transferred to the computer, and then, detection was started when the signals were stable. The base signal was initially collected for 5 minutes, followed by inflation for 5 minutes and data collection, then exhaustion immediately, and recording for 5 minutes to end the detection. Finally, the RHI of blood vessel was measured by software. The RHI was regarded as reference, and smaller RHI was indicative of more severe vascular endothelial dysfunction.

### 2.8 Radioimmunoassay

Twelve hours after the patients were on empty stomach, 2 mL of elbow venous blood was extracted in a recumbent position and then transferred to a tube with 30 µL of 10% ethylene diamine tetraacetic acid (EDTA) and 40 µL of trasylol, mixed well at 40°C, centrifuged for 10 minutes (3000 r/min) to separate blood plasma for preservation at −70°C. Before detection, the samples were thawed at a room temperature, centrifuged for 5 minutes (3000 r/min) at 4°C. The radioimmunoassay was conducted for serum detection. The reference value of ET‐1 was 50.82 ± 7.58 pg/mL.

### 2.9 Nitrate reductase assay (NRA)

Nitrate reductase assay detected the NO level in the blood. Venous blood on an empty stomach was extracted from four groups for NO detection. For the very reason that NO can rapidly transfer as nitrate and nitrite, nitrate can be deoxidized as nitrite in acidic environment or Cadmium reducing agent, and nitrite would present color reaction with Griess. According to the Griess kit, an appropriate amount of serum samples were added with Griess reagent, the absorbance value was measured by spectrophotometer at a wavelength of 550 nm, and NO level was calculated. The reagent with intra‐ and inter‐batch variation coefficient being 3% and 5%, respectively, was provided by Hongyang biotech company. NO level was detected within 24 hours of onset of LA and 8 weeks after ATV treatment on an empty stomach.

### 2.10 Follow‐up

Follow‐up was conducted by outpatient reexamination and telephone contact for recording the adverse reactions, such as gastrointestinal reaction, muscular pain, and increased aminopherase. Eight weeks after the treatment follow‐up was stopped, a total of 402 cases were followed up, with a rate of 100%.

### 2.11 Statistical analysis

The SPSS 19.0 software (IBM Corp.) was used for data analysis. The categorical data were presented in the form of number or percentage, and the chi‐square test was highlighted for comparisons among multiple groups. The measurement data were detected using normality test and homogeneity of variance test. One‐way analysis of variance (ANOVA) was conducted for comparisons among multiple groups, mean ± SD for data in normal distribution, t test for comparisons before and after ATV treatment, and Pearson correlation analysis for correlation analysis, with *P* < .05 signifying significant difference.

## 3 RESULTS

### 3.1 The clinical data of enrolled subjects

According to the different doses of ATV, all patients were allocated into the control (201 cases), ATV (10 mg, 67 cases), ATV (20 mg, 67 cases), and ATV (40 mg, 67 cases) groups. There were no remarkable differences in terms of age, gender, disease history, heart rate, blood pressure, and blood fat before ATV treatment (all *P > *.05), as shown in Table 1.

### 3.2 Patients in the control and ATV groups are at different grades of LA

**Figure 1:**
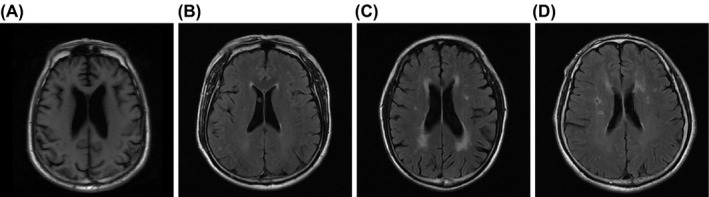
Patients in the control and ATV groups are at different grades of LA diagnosed by MRI images. A, MRI results of LA‐1 patients head; the lesion was confined to the white matter on the frontal or occipital horn of lateral ventricle; B, MRI results of LA‐2 patients head; the lesion was confined to the white matter on the frontal and occipital horn of lateral ventricle; C, MRI results of LA‐3 patients head; the lesion was confined to the white matter on the frontal horn, occipital horn, and body of lateral ventricle; D, LA‐4 patients head MRI; the lesion was confined to the white matter on the frontal horn, occipital horn, and central of the body of lateral ventricle; LA, leukoaraiosis; MRI, magnetic resonance imaging

MRI diagnosed patients in the four groups at different grades of LA. The case numbers of LA‐1, LA‐2, LA‐3, and LA‐4 patients in the control group were 98, 27, 29, and 29, respectively; the ATV (10 mg) group consisted of 65, 24, 19, and 21 cases at the LA‐1, LA‐2, LA‐3, and LA‐4 levels, respectively; the ATV (20 mg) group included 23, 12, 13, and 12 cases at the LA‐1, LA‐2, LA‐3, and LA‐4 levels, respectively, and the ATV (40 mg) group included 15, 4, 6, and 5 cases, at these four levels, respectively. No remarkable difference was observed in disease grade before ATV treatment (*P > *.05). LA presented long T1 or long T2 signal on MRI and T2WI images showed foci, unclear and unsharp border and high density of signals in T2FLAIR sequence demonstrated by water suppression MRIs, typically showing the following features: dot‐like abnormal high signal was observed in the deep white matter or basal ganglia, cap‐like abnormal high signal was observed in the anterior horn of the lateral ventricle, and patchy‐, strip‐, or ring‐like abnormal high signal was observed around white matter in the lateral ventricle. Generalized changes were also observed, namely the abnormal signals spread in a confluent way in the white matter area of brain, as shown in Figure 1.

### 3.3 ATV treatment reduces the levels of TC and LDL‐C in LA patients in a dose‐dependent manner

**Figure 2:**
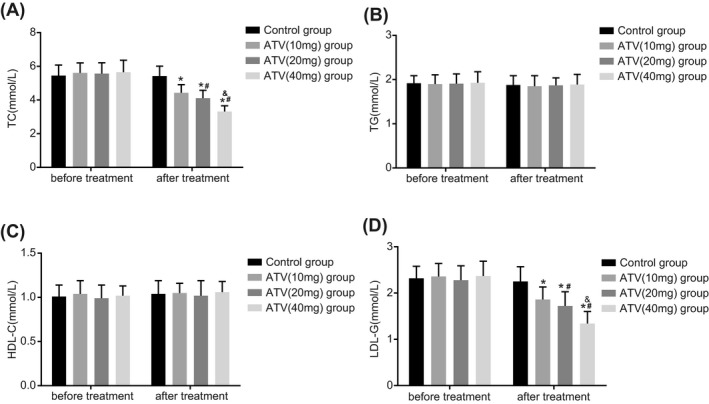
ATV treatment reduces the levels of TC and LDL‐C in LA patients in a dose‐dependent manner detected by enzyme colorimetric assay. A, comparisons of the TC level change in the patients of four groups; B, comparisons of the TG level change in the patients of four groups; C, comparisons of the HDL‐C level change in the patients of four groups; D, comparisons of the LDL‐C level change in the patients of four groups; *P < .05, compared with before ATV treatment; ^#^ *P* < .05, comparing the ATV (20 mg) and ATV (40 mg) groups with the ATV (10 mg) group; ^&^ *P* < .05, comparing the ATV (40 mg) group with the ATV (20 mg) group; ATV, atorvastatin; HDL‐C, high‐density lipoprotein cholesterol; LA, leukoaraiosis; LDL‐C, low‐density lipoprotein cholesterol; TC, total cholesterol; TG, triglycerides

Enzyme colorimetric assay was conducted to investigate whether ATV treatment with different doses could alter the levels of TC and LDL‐C in LA patients. Before ATV treatment, the TC, LDL‐C, HDL‐C, and TG did not highlight any significant difference in the four groups (*P > *.05). The levels of TC and LDL‐C decreased significantly 8 weeks after ATV treatment (*P < *.05). The levels of TC and LDL‐C decreased by 21% and 21.2%, respectively, in the ATV (10 mg) group while the levels decreased by 26.2% and 24.5%, respectively, in the ATV (20 mg) group along with a reduction in the levels by 41.2% and 43.4%, respectively, in the ATV (40 mg) group, highlighting a dose‐effect relationship of the higher dose of ATV with a higher decrease in the levels of TC and LDL‐C 8 weeks after ATV treatment. The levels of TG and HDL did not show any significant difference among ATV groups before and after ATV treatment (*P > *.05). All aforementioned indexes reduced significantly in the control group with no statistical differences (*P > *.05) (Figure 2). The results above demonstrated that ATV treatment could reduce the levels of TC and LDL‐C in LA patients in a dose‐dependent manner.

### 3.4 ATV treatment decreases the HS‐CRP level in LA patients in a dose‐dependent manner

**Figure 3:**
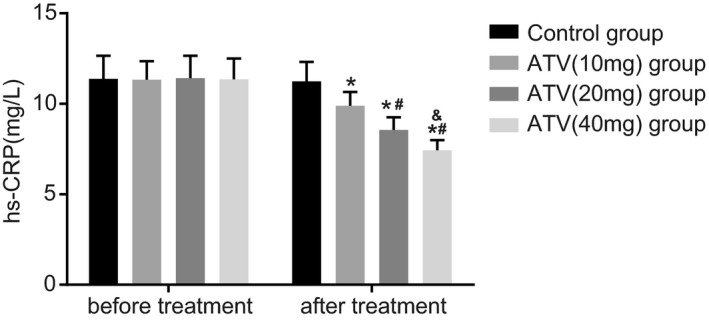
Latex agglutination test demonstrates that ATV treatment decreases the HS‐CRP level in LA patients in a dose‐dependent manner. *P < .05, compared with before ATV treatment; ^#^ *P* < .05, comparing the ATV (20 mg) and ATV (40 mg) groups with the ATV (10 mg) group; ^&^ *P* < .05, comparing the ATV (40 mg) group with the ATV (20 mg) group; ATV, atorvastatin; HS‐CRP, high‐sensitivity C‐reactive protein; LA, leukoaraiosis

In order to determine the HS‐CRP level in the control and ATV groups, latex agglutination test was employed. The HS‐CRP level decreased remarkably in the ATV (10 mg), ATV (20 mg), and ATV (40 mg) groups after 8 weeks of ATV treatment compared to before the treatment. The level decreased from 11.33 to 9.89 mg in the ATV (10 mg) group, while in the ATV (20 mg) group, the level dropped from 11.42 to 8.56 mg, and in the ATV (40 mg) group, the level dropped from 11.35 to 8.56 mg, which highlighted that in terms of HS‐CRP level, ATV (20 mg) group >ATV (40 mg) group >ATV (10 mg) group. In the control group, the HS‐CRP level dropped from 11.38 to 11.24, showing a relative low decreasing trend than that in the ATV groups (Figure 3). It was concluded that ATV treatment could decrease the HS‐CRP level in LA patients in a dose‐dependent manner.

### 3.5 ATV treatment elevates the levels of RHI, ET‐1, and NO in LA patients

**Figure 4:**
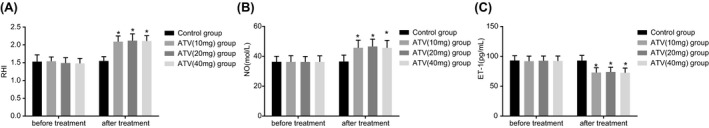
ATV treatment elevates the levels of RHI, ET‐1, and NO in LA patients detected by PAT technology, radioimmunoassay, and NRA. A, comparisons of the RHI level change in the patients of four groups before and after ATV treatment; B, comparisons of the NO level change in the patients of four groups before and after ATV treatment; C, comparisons of the ET‐1 level change in the patients of four groups before and after ATV treatment; *P < .05, compared with before ATV treatment; ET‐1, endothelin‐1; LA, leukoaraiosis; NO, nitric oxide; NRA, nitrate reductase assay; PAT, peripheral arterial tonometry; RHI, reactive hyperemia index

Subsequently, PAT technology, radioimmunoassay, and NRA were performed for determination of levels of RHI, ET‐1, and NO, respectively. As shown in Figure 4, there were no significant differences in the levels of RHI, ET‐1, and NO among the four groups (*P > *.05), while 8 weeks after ATV treatment, the levels of RHI and NO elevated remarkably in the three ATV groups (*P < *.05) and the levels also increased in the control group with no remarkable differences (*P > *.05) compared to those before treatment. The ET‐1 level reduced remarkably in the three ATV groups (*P < *.05) along with the control group with no remarkable difference (*P > *.05), which showed that different doses of ATV could significantly improve the RHI, ET‐1, and NO levels in LA patients to a different extent. All results above indicated that ATV could improve the vascular endothelial function within a dose of 10‐40 mg/d, signifying an insignificant correlation. It was suggested that ATV treatment could elevate the levels of RHI, ET‐1, and NO in LA patients, but this effect was independent of its dose.

### 3.6 RHI is negatively correlated with HS‐CRP and ET‐1 whereas exhibiting a positive correlation with NO level

**Figure 5:**
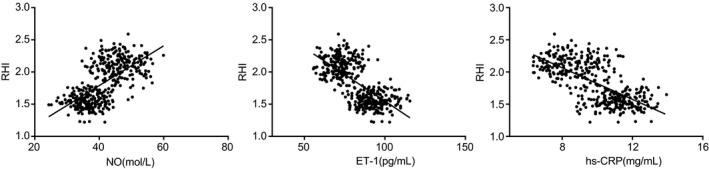
Pearson correlation analysis results demonstrate that RHI is negatively correlated with HS‐CRP and ET‐1 whereas exhibiting a positive correlation with NO level. A, the correlation between RHI and NO; B, the correlation between RHI and ET‐1; C, the correlation between RHI and HS‐CRP; ET‐1, endothelin‐1; HS‐CRP, high‐sensitivity C‐reactive protein; NO, nitric oxide; RHI, reactive hyperemia index

Pearson correlation analysis (Figure 5) was conducted to detect the correlation of RHI with HS‐CRP, ET‐1, and NO, which exhibited that the RHI was negatively correlated with HS‐CRP and ET‐1 (*r* = −.680 and −.678, both *P* < .05), whereas exhibiting a positive correlation with NO level (*r* = .647, *P* < .05).

### 3.7 Treatment with ATV (40 mg) contributes to the most severe adverse reactions

The adverse reactions experienced by all patients in the four groups were recorded. The records highlighted no adverse reaction in the control group, while four cases (6.0%) of gastrointestinal reaction, five cases (7.5%) of muscle pain, and three cases (4.5%) of transaminase elevation were observed in the ATV (10 mg) group; five cases (7.5%) of gastrointestinal reaction, 15 cases (22.38%) of muscle pain, and four cases (6.0%) of transaminase elevation were observed in the ATV (20 mg) group; and 14 cases (20.9%) of stomach upset, 26 cases (38.8%) of muscle pain, and 23 cases (34.3%) of transaminase elevation were observed in the ATV (40 mg) group. All transaminase levels in the four groups were less than three times of upper limit value, which indicated that all patients could still take the prescribed medicines (Table 2). The result showed that the muscle pain in the ATV (40 mg) and ATV (20 mg) groups was promoted significantly than the ATV (10 mg) group (*P* < .05), and all adverse reactions such as gastrointestinal reaction, muscle pain, and elevated transaminase were significantly obvious in the ATV (40 mg) group than in the ATV (20 mg) and ATV (10 mg) groups (*P* < .05). From the above results, treatment with ATV (40 mg) might result in the most severe adverse reactions in LA patients.

**Table 2:** Treatment with ATV (30 mg) contributes to the most severe adverse reactions

Group	Gastrointestinal reaction (n, %)	Muscle pain (n, %)	Transaminase elevation (n, %)
Control	0, 0%	0, 0%	0, 0%
ATV (10 mg) group	4, 6.0%	5, 7.5%	3, 4.5%
ATV (20 mg) group	5, 7.5%	15, 22.38%*	4, 6.0%
ATV (30 mg) group	14, 20.9%*, **	26, 38.8%*, **	23, 34.3%*, **

Abbreviation: ATV, atorvastatin.

* *P* < .05, compared with the ATV (10 mg) group;

** *P* < .05, compared with the ATV (20 mg) and ATV (30 mg) groups.

## 4 DISCUSSION

Leukoaraiosis, or white matter changes, is in close association with increased age, history of stroke, hypertension, and diabetes mellitus.^19^ It has been worldwide regarded as a part of the normal aging process, even though it is strongly associated with dementia or other disabilities, and its pathogenesis still has not been thoroughly acknowledged.^20^ Thus, our study examined the association between different doses of ATV and vascular endothelial function in patients with LA, aimed at providing a better therapeutic procedure for LA treatment. Finally, we concluded that different doses of ATV could have different effects on vascular endothelial function in patients with LA.

Initially, we found that the levels of TC, LDL‐C, and HS‐CRP decreased significantly in the ATV groups compared with the control group after 8‐week of ATV treatment, indicating that a proper dose of ATV could promote vascular endothelial function. Serum cholesterol, either total or lipoprotein fractions, is in closely association with human both mid‐ and late‐life depression.^21^ A cohort study was conducted for determining the relative risk and incidence for myocardial infarction, and it verified that TC was a significant risk factor for myocardial infarction.^22^ LDL‐C level is strongly associated with sustained viral response for chronic hepatitis C infection, and it was concluded that LDL‐C burden might identify those patients with pegIFN/RBV therapy.^23^ LDL‐C is also an well‐acknowledged indicator for primary care, and the Centers for Disease Control and Prevention (CDC)’s model can be employed for the analysis of quality indicators,^24^ which is also a traditional method for measurement of risk attributable to LDL.^25^ HS‐CRP is a biomarker for low‐grade inflammation, hyperglycemia, atherosclerosis, obesity, and hypertension.^26^ LDL and HS‐CRP have been regarded as high‐risk factors in vascular events, and 20 mg of atorvastatin had a remarkable effect on lipids.^27^ Remarkably, ATV‐induced increment in apelin was independently associated with changes in TC and LDL‐cholesterol.^28^ A study demonstrated that the ATV treatment had a close relation with remarkable reduction in target‐to‐background ratio (TBR) both in femoral aorta and in ascending artery, and TBR in both arteries associated with reductions in LDL‐C, MDA‐LDL‐C, and HS‐CRP.^29^ ATV was able to decrease LDL‐C and HS‐CRP in patients of European or South Asian origin.^30^ Most importantly, one study aimed at investigating the efficacy of ATV on levels of TC and HS‐CRP in atrial fibrillation (AF) patients in Asia suggested that ATV was significantly effective in lowering serum levels of HS‐CRP as well as TC so as to inhibit cardiovascular events.^31^ HS‐CRP, factor VII, and enhanced monocyte cytokine release were abnormalities, which could be attenuated by both ATV treatments.^32^ A combination of ATV with Berberine reduces levels of TC, TG, LDL‐C, and also oxidative stress and inflammation in rat models.^33^ Altogether, we concluded that decreased levels of TC, LDL‐C, and HS‐CRP in the ATV groups after 8‐week of ATV treatment promoted the vascular endothelial function.

Atorvastatin groups had increased NO level and RHI and decreased ET‐1 content compared with the control group after ATV treatment and severe complications such as upset stomach, muscular pain, and increased aminopherase. ET‐1, a potent vasoconstrictor, was secreted by vascular endothelial cells, consequently affecting the pathophysiology of various cardiovascular diseases.^34^ One study demonstrated that ET‐1 to some extent contributed to diminishing the endothelium‐dependent vasodilatation among older men.^35^ Moreover, NO and ET‐1 are natural parts in vascular function, and the imbalance between these two markers is a characteristic of endothelial dysfunction and is significant in vascular disease progression.^36^ The expression and effects of ET‐1 along with its receptors are significantly altered during the development of cardiovascular disease and the increased production of ET‐1 mediates many pathophysiological events contributing to the development of atherosclerosis and vascular complications in diabetes mellitus.^37^ RHI was calculated using RH‐PAT and was defined as the ratio of the digital pulse volume which could attenuate the severity in the patients with restenosis.^38^ Significantly, one study offered evidence that ATV was of significant importance in reducing ET‐1 level, which highlighted its antivasospastic effect on chronic vasospasm.^39^ In addition, another study found that compared to non‐ATV group, ET‐1 expression decreased in human umbilical vein endothelial cells of the ATV group but NO level increased, signifying that ATV could inhibit oxidative stress as well as endothelial damage.^40^ A prior study proved that liver ischemia‐reperfusion injury leads to a dramatic increase in the apoptosis and necrosis of hepatocytes, aminopherase activity, and production of pro‐inflammatory cytokines.^41^ Our findings were in line with the aforementioned studies; thus, we conclude that ATV treatment promoted vascular endothelial function in patients with LA.

To conclude, our study demonstrates that different doses of ATV can have different effects on vascular endothelial function in patients with LA, so as to provide a therapeutic way for LA treatment. However, there are limitations existing in this research. On the one hand, we did not observe that improving vascular endothelial function by atorvastatin correlated with its dosage significantly. Herein, it was necessary to expand the dose range of atorvastatin including the group of 80 mg/d. On the other hand, the polymorphism of apolipoprotein caused the inconformity of the statins drugs responses to lipid. In this study, we lacked the discussion on the effect of the polymorphism of apolipoprotein on the therapy of the statins drug. Hence, more investigations should be arranged in future.
